# Recent advances in de-intensification of radiotherapy in elderly cancer patients

**DOI:** 10.12688/f1000research.21151.1

**Published:** 2020-05-27

**Authors:** Isacco Desideri, Viola Salvestrini, Lorenzo Livi

**Affiliations:** 1 Department of Experimental and Clinical Biomedical Sciences Biochemistry, Radiotherapy Unit, University of Florence, Florence, Italy

**Keywords:** elderly, radiotherapy, de-intensification, prostate cancer, breast cancer, lung cancer

## Abstract

Cancer in the elderly remains an evolving issue and a health challenge. Several improvements in the radiotherapy field allow the delivery of higher doses/fractions with a safe toxicity profile, permitting the reduction of radiation treatment protocols in the elderly. Regarding breast, prostate, and lung cancer, the under-representation of older patients in clinical trials limits the extension of treatment recommendations to elderly patients in routine clinical practice. Among the feasible alternatives to standard whole breast radiotherapy (WBRT) in older patients are shorter courses using higher hypofractionation (HF) and accelerated partial breast irradiation (APBI). The boost continues to be used in women at high risk of local recurrence but is less widely accepted for women at lower risk and patients over 70 years of age. Regarding prostate cancer, there are no published studies with a focus on the elderly. Current management decisions are based on life expectancy and geriatric assessment. Regimens of HF and ultra-HF protocols are feasible strategies for older patients. Several prospective non-randomized studies have documented the safe delivery of ultra-HF for patients with localized prostate cancer, and multiple phase III trials and meta-analyses have confirmed that the HF regimen should be offered with similar acute toxicity regardless of patient age and comorbidity. A recent pooled analysis from two randomized trials comparing surgery to stereotactic body radiation therapy (SBRT) in older adult patients with early stage non-small cell lung cancer did show comparable outcomes between surgery and SBRT. Elderly cancer patients are significantly under-represented in all clinical trials. Thus, the inclusion of older patients in clinical studies should be strongly encouraged to strengthen the evidence base for this age group. We suggest that the creation of oncogeriatric coordination units may promote individualized care protocols, avoid overtreatment with aggressive and unrecommended therapies, and support de-escalating treatment in elderly cancer patients.

## Introduction

Cancer in the elderly population remains an evolving issue and a health challenge. Radiotherapy (RT) is an essential element of the management of older cancer patients ^1^. It is common knowledge to assess these patients based on functional status and not only on chronological age. Although the exact definition of the age group is controversial, we can define elderly patients as persons with a chronological age of 65 years and above ^2^. Whenever a decision has to be made regarding the intensity of treatment for elderly patients suffering with cancer, efforts should be focused on anticipating patients’ frailties leading to potentially harmful treatment side effects in this subset of patients. Comprehensive geriatric assessment (CGA) has been introduced by geriatricians to predict morbidity and mortality in elderly cancer patients. As the CGA requires the presence of a dedicated geriatrician and it is time-consuming, a number of different tools have been proposed as CGA surrogates to the scientific community. In a comprehensive review, the G8 was the most robust test, and it can be completed in less than 5 minutes ^3, 4^.

In an attempt to preserve elderly cancer patients’ quality of life with regard to toxicity while maintaining a curative intent, a tuning of treatment aggressiveness is a particularly attractive subject in this context. De-intensification of radiation treatment remains an unresolved problem. Novel RT approaches enable high doses to be administered safely to the tumor whilst lowering exposure to nearby critical organs. This has resulted in shorter curative and palliative protocols ^5^. Technological advances, such as intensity-modulated radiation therapy (IMRT) and image-guided radiation therapy (IGRT), have improved radiation effectiveness while reducing morbidity and improving daily patients’ set up and immobilization ^6– 8^.

Among improvements in RT delivery, efforts have been made to reduce toxicity related to organ motion, especially in the case of thoracic irradiation. Techniques limiting respiratory motion, such as controlled breath-hold and abdominal compression, are a viable option in the general cancer population; unfortunately, these approaches are frequently not tolerated by elderly patients. A feasible alternative could be the incorporation of 4D planning computed tomography (CT) scans. Other strategies to compensate for motion include respiratory gating, adjustment of field sizes, and tumor tracking ^9^. Ultimately, precise target definition allows better delineation of organs at risk and supports the delivery of higher doses/fractions with a safe toxicity profile, permitting the reduction of radiation treatment protocols in the elderly.

Despite the fact that the elderly are a growing significant proportion of cancer patients, they remain under-represented in most clinical studies of RT with limited level I evidence in this population. Regarding breast, prostate, and lung cancer, this under-representation of elderly patients in clinical trials limits the extension of treatment recommendations to this patient group in routine clinical practice. The aim of our work is to explain the principal strategies of de-intensification in elderly patients affected by different primary cancers obtainable through the adoption of these new RT techniques with a focus on curative indications.

## Breast cancer

Breast cancer is the most prevalent disease in elderly women and the primary cause of death. Management is undefined in this patient group because elderly women are rarely included in relevant clinical trials ^10^. The gold standard for all patients affected by early invasive breast cancer (IBC) is whole breast irradiation (WBI) following breast-conserving surgery (BCS). The Oxford overview regarding trials on BCS with or without WBI highlights a decrease in first recurrence in “low-risk” older patients; even if comparing the elderly population (70 years of age or older) with younger patients, the absolute 10-year risk reduction of any locoregional or distant relapse is lower ^11^. A randomized study (the CALGB 9343 trial) evaluating 636 older women (aged 70 years) with IBC treated with BCS and adjuvant tamoxifen with or without WBI showed an absolute decrease in ipsilateral breast tumor recurrence (IBTR) rate of 3% at 5-year follow-up and of 7% in the RT group ^12^.

Among the feasible treatment modalities for older patients, shorter courses using hypofractionated (HF) schedules are available. Several randomized controlled trials (RCTs) have pointed out similar rates of local control and late toxicity in terms of cosmesis using heterogeneous HF-RT regimens after BCS ^13, 14^. In light of recent findings, the 2018 ASTRO WBI guidelines endorse HF-WBI in patients with breast cancer irrespective of their age, the stage of their tumor, and whether or not they have undergone chemotherapy ^15^.

The rationale for the delivery of boost to the tumor bed in patients receiving BCS and WBI comes from evidence that local relapse is commonly found at the site of the primary tumor. In detail, 44 to 90% of local recurrences occurred in proximity of the tumor bed ^16, 17^. Several randomized studies compared irradiation with or without tumor bed boost in a timeframe of about 10 years ( Table 1) ^18– 21^. Findings obtained from these five studies confirmed that delivery of boost after WBI offered a benefit in terms of decrease in local relapses, without any detrimental effect on survival or other outcomes ^22^. Furthermore, the impact of bed boost diluted as the patient’s age increased ^21^. Among reasons for questioning the boost, we identified higher treatment costs and potentially adverse events. The size of the absolute benefit of the boost dose for tumor control decreases with increasing age; this limited gain in local control in older patients should be weighed against the increase in risk of late adverse effects such as fibrosis ^21, 23, 24^. According to current standards, omitting a tumor bed boost is suggested with conditional recommendation strength and moderate quality of evidence in patients with IBC who are older than 70, are hormone receptor positive, and have tumors of low or intermediate grade resected with widely negative (≥2 mm) margins ^15^.

**Table 1.** Phase III trials investigating tumor bed boost after breast-conserving surgery.

Trial	Study period	Study patients, overall (elderly)	Elderly patients (%)	RT technique, study design	OS rates	LR rates	Subgroup analysis in elderly
**EORTC ^21^**	1989–1996	5,318 (1,732)	32.6 (>60 years)	EBRT; LDR brachytherapy ° WBI vs. WBI+boost	At 20 years: 61.1% (WBI) vs. 59.7% (WBI+boost); *P* = 0.33	At 10 years: 13% (WBI) vs. 9% (WBI+boost); *P* <0.0001	Yes
**Lyon ^18^**	1986–1992	1,024 (272)	26.5 (>61 years)	EBRT ° WBI vs. WBI+boost	At 5 years: 90.4% (WBI) vs. 92.9% (WBI+boost); *P* = 0.24	At 3.3 years: 4.5% (WBI) vs. 3.6% (WBI+boost); *P* = 0.44	No
**Budapest ^19^**	1995–1998	207 (NR)	NR	EBRT; HDR brachytherapy ° WBI vs. WBI+boost	NR	At 5 years: 5.1% (WBI) vs. 7.3% (WBI+boost); *P* = 0.049	No
**SWG ^20^**	1996–NR	674 (NR)	NR	EBRT ° WBI vs. WBI+boost	NR	At 8.5 years: 2% (WBI) vs. 4.4% (WBI+boost); *P* = NR	No

EBRT, external beam radiotherapy; HDR, high-dose rate brachytherapy; LDR, low-dose-rate brachytherapy; LR, local relapse; NR, not reported; OS, overall survival; RT, radiotherapy; WBI, whole breast irradiation. °Tumor bed boost techniques

Accelerated partial breast irradiation (APBI), combining increased dose per fraction, short duration of treatment, and small target volume confined to the tumor bed, represents an attractive de-escalating strategy in elderly women. The role of partial breast irradiation (PBI) has been investigated in large-scale prospective phase III clinical trials ( Table 2) ^25– 29^. The main recommendations published by the European Society for Radiotherapy and Oncology (ESTRO) ^30^ and the American Society for Radiotherapy and Oncology (ASTRO) ^31^ apply a specific age cut-off for defining patients who are suitable for PBI. Particularly, PBI is recommended only in patients older than 50. Regarding selected older patients (≥50 years, low tumor grade, up to 3 cm diameter, positive status of estrogen receptor, and HER2-negative status without nodal involvement), the 2016 UK consensus statements on breast cancer adjuvant irradiation recommended choosing external beam RT (EBRT) or multiple catheter brachytherapy PBI ^32^. Single fraction intra-operative RT (IORT) has been tested in two large phase III RCTs with conflicting results, making its recommendation for this subset of patients still controversial ^28, 29^.

**Table 2.** Phase III trials investigating partial breast irradiation.

Trial	Study period	Study patients, overall (elderly)	Elderly patients (%)	RT technique, study design	OS rates	LR rates	Subgroup analysis in elderly
**IMRT APBI** **Florence ^25^**	2005–2013	520 (117)	22.5 (>o = 70 years)	Accelerated IMRT APBI vs. WBI	At 5-year: 99.4% (APBI) vs. 96.6% (WBI); *P* = 0.057	At 5-year: 1.5% (APBI) vs. 1.5% (WBI); *P* = 0.86	Yes
**GEC-ESTRO ^26^**	2004–2009	1,184 (190)	16 (>70 years)	Brachytherapy APBI vs. WBI	At 5-year: 97.27% (APBI) vs. 95.55% (WBI); *P* = 0.11	At 5-year: 1.44% (APBI) vs. 0.92% (WBI); *P* = 0.42	No
**IMPORT** **LOW ^27^**	2007–2010	2,018 (NR)	NR	Normofractionated IMRT PBI vs. WBI	At 5-year: 3.7% (PBI) vs. 5% (WBI); *P* = 0.693	At 5-year: 0.5% (PBI) vs. 1.1% (WBI); *P* = 0.420	No
**TARGIT-A ^28^**	2000–2012	3,451 (NR)	NR	IORT ° IORT vs. WBI	At 5-year: 96.1% (IORT) vs. 94.7% (WBI); *P* = 0.099	At 5-year: 3.3% (IORT) vs. 1.3% (WBI); *P* = 0.042	No
**ELIOT ^29^**	2000–2007	1,305 (137)	10.5 (>o = 70 years)	IORT ° IORT vs. WBI	At 5-year: 96.8% (IORT) vs. 96.9% (WBI); *P* = 0.59	At 5-year: 4.4% (IORT) vs. 0.4% (WBI); *P* <0.0001	Yes

APBI, accelerated partial breast irradiation; CI, confidence interval; EBRT, external beam radiotherapy; IBTR, ipsilateral breast tumor recurrence; IMRT, intensity modulated radiotherapy; IORT, intraoperative radiotherapy; LR, local relapse; NR, not reported; OS, overall survival; PBI, partial breast irradiation; RT, radiotherapy; WBI, whole breast irradiation. °Experimental arm technique

IORT has several limitations, such as the lack of certain margin, the absence of image-guided treatment planning, and the use of low-energy photons. In order to minimize these aspects, Showalter *et al*. tested CT-based treatment planning and high-dose-rate (HDR) brachytherapy (CT-HDR-IORT). This novel technique permits 3D treatment planning, demonstrating a favorable feasibility and safety profile in women with early breast cancer. Currently, a phase II trial on CT-HDR-IORT is underway ^33^.

To date, only one dedicated paper has been published regarding the outcome of elderly patients undergoing PBI in a RCT. The authors’ subgroup analysis included elderly (≥70 years) women with early low-risk breast cancer (hormone receptor-positive, axillary node-negative, T1–T2 up to 3 cm, and clear margins). In this subpopulation, the reduction of overall treatment duration, consisting of a dose of 30 Gy in five non-consecutive daily fractions (2 weeks of treatment), led to a better quality of life profile and no significant difference in terms of IBTR compared with WBI ^34^.

In order to minimize the days of therapy schedules, many authors focused their efforts on different regimens of PBI, and several trials have been designed to evaluate novel HF APBI schedules ^35– 40^. Wilkinson *et al*. ^35^ collected data from 45 patients undergoing adjuvant APBI in 2 days with HDR brachytherapy. The local, regional, and distant control were in line with literature, and toxicity was acceptable. In Latorre *et al*.’s analysis, considering a single 18 Gy fraction with multi-catheter HDR brachytherapy, none of the 20 patients reported grade three toxicity ^36^. According to Jethwa *et al*., the three-fraction once-daily intracavitary catheter-based partial breast brachytherapy (ICBB) presents a low rate of early provider and patient-reported adverse events and a favorable breast cosmesis at early follow-up ^37^. In addition, a three-fraction, phase II APBI trial (TRIUMPH: TRI-fraction Radiotherapy Utilized to Minimize Patient Hospital Trips) confirmed that ultrashort breast brachytherapy can be delivered with low toxicity and a good short-term tolerance profile ^38^. Compared with traditional 5-day treatment, these novel schedules of adjuvant RT have an excellent local control for early stage IBC and an acceptable toxicity profile, but they were not tested in elderly subpopulations and this patient group’s selection continues to be challenged. With this in mind, a retrospective analysis of elderly patients (≥65 years old) confirmed the excellent toxicity profile and clinical outcome of a postoperative single fraction of APBI ^39^. According to Hannoun-Lévi *et al*.’s data from 26 elderly patients treated with APBI (HDR brachytherapy), the clinical outcomes remain optimal and the toxicity profile was very low (with 15.4% and 3.9% of grade one and grade two toxicity, respectively) ^40^. In order to evaluate these two important endpoints and confirm these advantages in the elderly population, additional RCTs of HF APBI are needed.

In women aged 70 or older with low-risk, hormone-positive breast cancer, the literature and clinical practice focused efforts to escalate endocrine therapy (ET) and to de-escalate chemotherapy. However, the independent contribution of ET and chemotherapy on the quality of life is not clarified. Ferreira *et al*. reported that ET had a major detrimental effect on C30 summary score (C30-SumSc) at 2 years after diagnosis, especially in postmenopausal women. These results suggest that radiation therapy alone should be considered for older women with early stage, biologically favorable breast cancer ^41, 42^. Notably, Buszek *et al*. showed that for healthy, older women with biologically favorable disease, adjuvant irradiation or ET is related to similar 5-year overall survival (OS) rates. Thus, in order to evaluate monotherapy strategy, RCTs are needed ^43, 44^.

Finally, the side effects of RT alone or when combined with other therapies are an important issue in elderly patients because of their impact on quality of life. To our knowledge, clinical guidelines recommend at least 5 years of treatment with adjuvant hormone therapy to prevent local relapse and improve survival ^45^. However, the toxicity profile of ET is relevant, specifically for the elderly, and ET adherence rates decline among women over 70 ^43– 46^.

On the other hand, adjuvant breast irradiation presents an excellent tolerance profile. The main side effects, skin reactions, edema, and fibrosis, do not significantly affect quality of life and patients’ daily activities. Moreover, radiation therapy adherence rates range from 98 to 99% and treatment period from 1 to 5 weeks, suggesting that it is potentially more suitable for the elderly population ^47^.

## Prostate cancer

The median age at prostate cancer (PC) diagnosis is 66 years, and 69% of deaths occur in men aged 75 ^48^. Recent data suggest that significant comorbidity in elderly patients older than 75 should be a relative contraindication to aggressive treatment in low-risk PC ^49^; there are no published studies focused solely on the elderly. Current management decisions are based on life expectancy and geriatric assessment.

Men with clinically localized PC, particularly with low-risk disease, have extremely low rates of cancer-specific mortality within 10 years of diagnosis. Among the feasible treatment strategies there are active surveillance (AS) and watchful waiting (WW). The distinction between these approaches is important for clinical decision-making. AS, which carries a curative intent and involves regular monitoring with prostate-specific antigen, digital rectal examination, and biopsy, is appropriate for patients who have sufficient life expectancy to benefit from active treatment if disease progression is detected. On the other hand, for patients with a life expectancy of less than 5 years, WW (cessation of routine monitoring with treatment initiated only if symptoms develop) is appropriate and further reduces the issue of overtreatment in PC ^50^.

When RT is selected, androgen deprivation therapy (ADT) benefit is still unproven. In the RTOG 94-08 trial, 10-year survival in the low-risk group was similar after EBRT with or without ADT ^51^. In men with low-risk PC who decline AS and choose active treatment with EBRT, HF and extreme HF, consisting of fraction size ≥500 cGy (typically with a maximum number of five fractions), may be offered as an alternative to conventional fractionation. According to the current state of the art, there are no RCTs reporting efficacy and toxicity data on the comparison between ultra-HF and conventionally fractionated schedules. However, several prospective non-randomized studies have confirmed the safety of ultra-HF for localized PC patients; these findings seem to be applicable to patients without severe urinary symptoms and with up to 100 cm ^3^ of prostate volume at baseline. Currently, several trials are ongoing or in design ( Table 3) ^52^.

**Table 3.** Randomized trials evaluating ultra-hypofractionated external beam radiotherapy in prostate cancer.

Trial	Planned accrual	Elderly patients (%)	Cancer risk groups	Primary endpoint	Ultra- hypofractionated arm	Comparator arm	Status
**HEAT** **NCT01794403**	456	NR	LR and IR	Biochemical or clinical failure	36.25 Gy in five fractions	70.20 Gy in 26 fractions	Accruing
**HYPO-RT-PC** **ISRCTN45905321**	1,200	NR	IR	Biochemical or clinical failure	42.7 Gy in seven fractions	78 Gy in 39 fractions	Accrual complete
**NRG-GU005**	606	NR	IR	Health-related quality of life toxicity assessment	36.25 Gy in five fractions	70 Gy in 28 fractions	Accruing
**PACE B** **NCT01584258**	858	NR	LR and IR	Biochemical or clinical failure	36.25 Gy in five fractions	78 Gy in 39 fractions or 62 Gy in 20 fractions	Accrual complete

IR, intermediate risk; LR, low risk; NR, not reported.

In intermediate-risk patients, large randomized trials ^51^ showed that the addition of hormones to EBRT resulted in increased survival ^53– 55^. Finally, in high-risk PC patients, combined therapy is the gold standard established by the Early Prostate Cancer Program and EORTC trials ^56^. Regarding the chance of reduction of treatment in the elderly, Bekelman et al. showed that in locally advanced PC patients (over 75 years of age), ADT with RT was associated with reduced cause-specific and all-cause mortality, and it remains the gold standard of therapy for PC patients of all ages ^57^.

Concerning radiation treatment de-intensification in terms of overall treatment time, multiple phase III RCTs and meta-analyses have compared oncologic outcomes, toxicity, and quality of life for moderate HF versus conventional fractionation. Moderate HF has the advantage of shortening treatment duration and improving cost-effectiveness with a gain in terms of patient compliance, especially in the elderly population ( Table 4) ^58– 64^. In patients who are candidates for EBRT, the HF regimen should be offered with similar acute toxicity regardless of the patient’s class of risk, age, and comorbidities ^52^.

**Table 4.** Randomized trials evaluating hypofractionated regimen.

Trial	Study period	Study patients, overall (elderly)	Elderly patients (%)	Median FUP	Study design	Study arms	RT technique	Cancer risk groups	ADT use and duration (median)	Age (median)	Disease control (hazard ratio – 1 ° endpoint)	Subgroup analysis in elderly
**CHHiP ^58^**	2002–2011	3,216 (1,615)	50.2 (>69 years)	5.2 years	Multicenter non- inferiority trial 1 ° endpoint: biochemical or clinical failure-free rate	74 Gy in 37 fractions 60 Gy in 20 fractions 57 Gy in 19 fractions	IMRT IGRT optional (53% use)	12% HR 15% LR 73% IR	97% 3–6 months (median 5.5 months)	68 years	60 vs. 74 Gy: 0.83 (90% CI: 0.64–1.14) 57 vs. 74 Gy: 1.20 (90% CI: 0.99–1.46)	Yes
**HYPRO ^59^**	2007–2010	820 (411)	50.1 (>70 years)	5 years	Multicenter superiority 1 ° endpoint: relapse-free survival	78 Gy in 39 fractions 64.6 Gy in 19 fractions	IMRT IGRT	26% IR 74% HR	67% Variable duration (median 32.4 months)	71 years	0.86 (95% CI: 0.63–1.16)	Yes
**PROFIT ^60^**	2006–2011	1,206 (NR)	NR	6 years	Multicenter non- inferiority trial 1 ° endpoint: biochemical or clinical failure	78 Gy in 39 fractions 60 Gy in 20 fractions	IMRT or 3D CRT IGRT required	100% IR	None	71 years	0.99 (90% CI: 0.83–1.19)	No
**RTOG** **0415 ^61^**	2006–2009	1,115 (420)	37.7 (> o =70 years)	5.8 years	Multicenter non- inferiority trial 1 ° endpoint: disease-free survival	73.8 Gy in 41 fractions 70 Gy in 28 fractions	IMRT or 3D CRT IGRT required	100% LR	None	67 years	0.85 (95% CI: 0.64–1.14)	No
**Fox** **Chase ^62^**	2002–2006	303 (NR)	NR	5.7 years	Single institution superiority trial 1 ° endpoint: biochemical disease-free survival	76 Gy in 38 fractions 70.2 Gy in 26 fractions	IMRT u/s IGRT	66% IR 33% HR	46% (median NR)	NR	1.38 (95% CI: 0.79–2.40)	No
**MD** **Anderson ^63^**	2001–2010	206 (82)	39.8 (> o =70 years)	5 years	Single institution superiority trial 1 ° endpoint: failure- free survival	75.6 Gy in 42 fractions 72 Gy in 30 fractions	IMRT or IGRT	28% LR 71% IR 1% HR	24% <4 months (median not reported)	67 years	NR	No
**Italian ^64^**	2003–2007	168 (NR)	NR	9 years	Single institution superiority trial 1 ° endpoint: late toxicity	80 Gy in 40 fractions 62 Gy in 20 fractions	3D CRT	100% HR	100% 9 months (median NR)	75 years	0.62 (95% CI: 0.34-1.14)	Yes

1°, primary; ADT, androgen deprivation therapy; CI, confidence interval; CRT, conformal radiation therapy; EBRT, external beam radiotherapy; FUP, follow up; HR, high risk; IGRT, image-guided radiation therapy; IMRT, intensity modulated radiotherapy; IR, intermediate risk; LR, low risk; NR, not reported; RT, radiotherapy.

A SIOG PC task force has updated recommendations for the management of elderly men with PC showing that these patients should be managed according to their health status and not according to age, although the extrapolation specifically to older patients is not straightforward. For these reasons, new clinical trials targeting the elderly population are needed ^65^.

Finally, radiation treatment for localized PC is associated with potential side effects, particularly in the elderly population. In EBRT, acute adverse events are typically not severe and resolve within 4–8 weeks after treatment; regarding long-term side effects, proctitis with bloody stools are the most common late rectal toxicities ^66^. Compared with radical prostatectomy, aside from a worse performance when considering quality of life in the bowel domain, RT is associated with better quality of life regarding urinary and sexual disorders ^67^. In addition, patients undergoing pelvic RT present an increased risk for fractures, spontaneously or after minimal trauma. In light of this, Vitztum *et al*. evaluated 28,354 patients aged over 65 undergoing RT for pelvic malignancies and found that IMRT and brachytherapy were associated with a reduced risk of pelvic fractures. Pelvic insufficiency fracture risk should be considered when treating old PC patients with radiation treatments ^68^. On the other hand, the use of ADT is not without adverse events. A recent analysis of 31 patients highlighted the high prevalence of muscle disorders, such as sarcopenia, before the initiation of ADT in a population of elderly PC patients ^69^. The optimal strategy for the elderly should be based on all of the aforementioned issues, but the correct patient selection continues to be challenged and additional studies in this subpopulation are needed.

## Lung cancer

Elderly cancer patients are significantly under-represented in all clinical trials, including lung cancer studies. ASTRO recommends a management strategy involving multiple disciplines and patients and physicians sharing the decision-making in early stage non-small cell lung cancer (NSCLC). Current recommendations include the use of stereotactic body RT (SBRT) in patients considered to be high risk for surgery, including those with either forced expiratory volume at 1 second or diffusing capacity of the lungs for carbon monoxide less than 50% predicted or a combination of age, reduced lung function, pulmonary hypertension, and low left ventricular function ^70^.

Insights regarding the utilization and survival of surgery and SBRT or conventional RT are lacking for older patients with stage I and II NSCLC in clinical practice. Because of limited accrual, a number of randomized trials evaluating surgery compared to SBRT in early stage operable patients have closed early. Nevertheless, a recent pooled analysis, based on STARS and ROSEL trials, did point out comparable local control and OS rates between surgery and SBRT. This approach represents a feasible, non-invasive treatment strategy for elderly patients with early stage NSCLC ( Table 5) ^71^. There are several retrospective studies examining outcomes between SBRT and surgery in older adult patients with early stage NSCLC ( Table 6) ^72^. Miyazaki *et al.* evaluated 98 patients with early stage NSCLC who were 80 years of age or older and who underwent SBRT or resection. Toxicities were similar and, after propensity score matching allowed for balanced patient characteristics, there were no significant differences in 5-year OS or disease-specific survival between the two treatments ^73^. Wang *et al.* performed a similar study and patients undergoing surgery were younger than populations undergoing SBRT (median age 72 versus 82), were in better health, and had better Eastern Cooperative Oncology Group (ECOG) performance status scores. According to propensity-score matching, surgery, compared to SBRT, still showed better locoregional relapse and recurrence-free survival with no significant results in terms of OS and disease-specific survival in this subset ^74^. Palma *et al.* evaluated patients 75 years of age or older with early stage NSCLC between 2005 and 2007. Comparison between 60 patients undergoing SBRT and 60 patients treated with surgery showed that 1- and 3-year OS rates were not significantly different and that 30-day mortality was 8.3% and 1.7% for surgery and SBRT, respectively ^75^. All of these retrospective studies suggest SBRT may be a feasible and effective treatment modality in comparison to surgical resection despite the fact that further prospective data are needed, specifically in elderly patients.

**Table 5.** Stereotactic ablative radiotherapy versus lobectomy for operable stage I non-small cell lung cancer: two randomized trials.

Trial	Study period	Study patients, overall (elderly)	Elderly patients (%)	RT technique, study design	OS rates	LR rates	Subgroup analysis in elderly
**STARS and** **ROSEL trials** **^71^** **[STARS:** **NCT00840749;** **ROSEL:** **NCT00687986]**	2008–2013	STARS: 36 (NR); ROSEL: 22 (NR)	STARS: NR ROSEL: NR	STARS CyberKnife SABR 60 Gy in four fractions or 60 Gy in three fractions vs. surgery (thoracotomy and VATS) ROSEL SABR 54 Gy in three fractions or 60 Gy in five fractions vs. surgery	3-year OS (95% CI): SABR 95% (85–100); surgery 79% (64–97) HR (95% CI): 0.14 (0.017–1.190) *P* = 0.037	3-year RFS (95% CI): SABR 86% (74–100); surgery 80% (65–97) HR (95% CI): 0.69 (0.21–2.29) *P* = 0.5379	No

CI, confidence interval; HR, hazard ratio; LR, local recurrence; NR, not reported; OS, overall survival; RFS, recurrence-free survival; SABR, stereotactic ablative radiotherapy; RT, radiotherapy; VATS, video-assisted thoracotomy.

**Table 6.** Retrospective studies examining outcomes between stereotactic body radiotherapy and surgery in older adult patients with early stage non-small cell lung cancer.

Trial	Study period	Study patients, overall (elderly)	Elderly patients (%)	RT technique, study design	OS rates	LR
**Miyazaki *et al*. ^73^**	2008–2014	98 (98)	100 (>80 years)	SBRT (48 Gy in four fractions) vs. surgery (lobectomy)	5-year OS with SBRT 68.3% (n = 57) vs. 47.4% (n = 41), HR=2.46 (95% CI 1.18–5.48), *P* = 0.02	NR
**Wang *et al*. ^74^**	2002–2010	180 (100)	55.5 (>75 years)	SBRT (60 Gy in five fractions)	3-year OS with SBRT 54.9%	3-year LRC with SBRT: 68.8%
**Palma *et al*. ^75^**	2005–2007	346 (346)	100 (>o = 75 years)	SBRT (60 in three, five, or eight fractions) vs. surgery (lobectomy, pneumonectomy or sublobar excision)	3-year OS with SBRT 42%	NR

CI, confidence interval; HR, hazard ratio; LC, local control; LR, local recurrence; LRC, locoregional control; NR, not reported; OS, overall survival; RT, radiotherapy; SBRT, stereotactic body radiotherapy.

According to current standards, local treatment, such as SBRT, is not reasonable for more advanced NSCLC; combined RT to the primary and involved nodes with chemotherapy tailored to comorbidities is the correct treatment modality in selected inoperable patients. In older patients undergoing surgical resection, efforts should focus on selecting an appropriate postoperative treatment for positive margins or pN2 disease ^76^.

Typically, RT has been associated with chemotherapy to improve outcome; with the introduction of immune checkpoint inhibitors as an effective treatment strategy, the evaluation of outcomes of concomitant RT and immunotherapy becomes mandatory ^77^. A lot of retrospective studies reported that radiation treatment has the potential to enhance the effects of checkpoint inhibition in selected patients. Similarly, some preclinical studies described a synergic effect of combination radiation–immunotherapy ^78^. Currently, numerous RCTs testing the combination of immunotherapy and radiation are ongoing ^79, 80^. Data from the PACIFIC trial, a phase III randomized study, evaluated consolidation therapy after chemoradiation for stage III NSCLC with the anti-PDL1 durvalumab versus placebo. The results confirmed the benefit of durvalumab in progression-free survival (PFS) while preserving a similar toxicity profile ^81^.

Regarding limited-disease small-cell lung cancer (LD-SCLC), phase III trials, in which older patients were as usual excluded, show that the standard treatment strategy remains platinum-based multiagent chemotherapy and thoracic and cranial irradiation ^82^. De-intensification of treatment in this setting of elderly patients has been investigated through diminutions in time and intensity of both treatments with heterogeneous findings, and standard chemoradiation is still suitable for fit older patients. The comparison between conventional (66 Gy) and accelerated (45 Gy b.i.d.) fractionation with standard platinum-based chemotherapy (cisplatin/etoposide) in patients of all ages (the phase-III CONVERT trial) highlights that survival outcomes did not differ between twice-daily and once-daily concurrent chemoradiotherapy, with a similar toxicity profile. Since the study was not powered to confirm equivalence between different treatment protocols, the conclusion is that twice-daily irradiation remains the standard therapy in this population ^83, 84^.

For both locoregionally advanced NSCLC and LD-SCLC, the investigation of concomitant treatment strategies incorporating newer chemotherapy and targeted agents, potentially less toxic than current cisplatin-based therapy, is warranted, especially in elderly patients.

## Conclusion

The evidence discussed herein emphasizes the need to more thoroughly research the elderly patient population, a group that is frequently overlooked in treatment guidelines. The complex heterogeneity of this patient group, in terms of age, comorbidities, and the presence of a caregiver, may warrant the historical exclusion of older patients from randomized trials. CGA and other surrogates such as G8 have not been fully implemented both in academic and in everyday practice. Lastly, the inclusion of older patients in clinical studies should be strongly encouraged to strengthen the evidence base for this age group. In this regard, we recommend a multidisciplinary approach based on the collaboration among surgeons, radiation oncologists, medical oncologists, and geriatricians. The creation of oncogeriatric coordination units may promote individualized care protocols, avoid overtreatment with aggressive and unrecommended therapies, and support de-escalating treatment in elderly cancer patients.

## Abbreviations

ADT, androgen deprivation therapy; APBI, accelerated partial breast irradiation; AS, active surveillance; ASTRO, American Society for Radiotherapy; BCS, breast-conserving surgery; CGA, comprehensive geriatric assessment; CT, computed tomography; EBRT, external beam radiation therapy; ET, endocrine therapy; HDR, high dose rate; HF, hypofractionation; IBC, invasive breast cancer; IBTR, ipsilateral breast tumor recurrence; IMRT, intensity-modulated radiation therapy; IORT, intra-operative radiotherapy; LD-SCLC, limited-disease small-cell lung cancer; NSCLC, non-small cell lung cancer; OS, overall survival; PBI, partial breast irradiation; PC, prostate cancer; RCT, randomized controlled trial; RT, radiotherapy; SBRT, stereotactic body radiotherapy; WBI, whole breast irradiation; WW, watchful waiting. 
